# The Association Between Branched-Chain Amino Acid Concentrations and the Risk of Autism Spectrum Disorder in Preschool-Aged Children

**DOI:** 10.1007/s12035-024-03965-4

**Published:** 2024-01-24

**Authors:** Qi Gao, Dan Bi, Bingbing Li, Min Ni, Dizhou Pang, Xian Li, Xiaoli Zhang, Yiran Xu, Qiang Zhao, Changlian Zhu

**Affiliations:** 1 Key Clinical Laboratory of Henan Province, Department of Clinical Laboratory, First Affiliated Hospital of Zhengzhou University, Zhengzhou, 450052 China; 2 Department of Pediatrics, Qilu Hospital of Shandong University, No. 107, Wen Hua Xi Road, Jinan, 250012 Shandong China; 3 Henan Key Laboratory of Child Brain Injury and Henan Pediatric Clinical Research Center, Third Affiliated Hospital and Institute of Neuroscience of Zhengzhou University, Zhengzhou, 450052 China; 4 Department of Henan Newborn Screening Center, The Third Affiliated Hospital of Zhengzhou University, Zhengzhou, 450054 China; 5 Center for Child Behavioral Development, Third Affiliated Hospital of Zhengzhou University, Zhengzhou, 450052 China; 6 Henan Key Laboratory of Children’s Genetics and Metabolic Diseases, Children’s Hospital Affiliated to Zhengzhou University, Zhengzhou, China; 7 Center for Brain Repair and Rehabilitation, Institute of Neuroscience and Physiology, University of Gothenburg, 40530 Gothenburg, Sweden

**Keywords:** Branched-chain amino acids, Autism spectrum disorders, Nomogram, Early diagnosis, Preschool children

## Abstract

Several studies have linked branched-chain amino acid (BCAA) metabolism disorders with autism spectrum disorder (ASD), but the results have been inconsistent. The purpose of this study was to explore the association between BCAA concentrations and the risk of ASD. A total of 313 participants were recruited from two tertiary referral hospitals from May 2018 to July 2021. Concentrations of BCAAs in dried blood spots were analyzed using liquid chromatography-tandem mass spectrometry-based analysis. Multivariate analyses and restricted cubic spline models were used to identify the association between BCAAs and the risk of ASD, and a nomogram was developed by using multivariate logistic regression and the risk was determined by receiver operating characteristic curve analysis and calibration curve analysis. Concentrations of total BCAA, valine, and leucine/isoleucine were higher in the ASD group, and all of them were positively and non-linearly associated with the risk of ASD even after adjusting for potential confounding factors such as age, gender, body mass index, and concentrations of BCAAs (*P* < 0.05). The nomogram integrating total BCAA and valine showed a good discriminant AUC value of 0.756 (95% CI 0.676–0.835). The model could yield net benefits across a reasonable range of risk thresholds. In the stratified analysis, the diagnostic ability of the model was more pronounced in children older than 3 years. We provide evidence that increased levels of BCAAs are associated with the risk of ASD, and the nomogram model of BCAAs presented here can serve as a marker for the early diagnosis of ASD.

## Background

Autism spectrum disorder (ASD) presents with a wide array of symptoms characterized by impaired social interaction, deficits in communication, and restricted and stereotyped behaviors and interests [1]. ASD is a complex disorder of unknown etiology, and understanding the pathogenesis of ASD is the key to developing therapies for its prevention and treatment. While genetic factors are believed to play an important role, many environmental factors have been associated with ASD [2].

Branched-chain amino acids (BCAAs), which include leucine/isoleucine (Leu) and valine (Val), are essential amino acids that comprise as much as 30% of all proteins. The brain is dependent on a constant supply of BCAAs from the periphery [3], and BCAA homeostasis is critical for the proper function of the central nervous system. BCAAs are metabolized through a multistep process, leading to the formation of acetoacetate, acetyl-Coenzyme A, and succinyl-Coenzyme A, which are critical for ATP production [4]. Both the accumulation and deficiency of BCAAs have been associated with different neurological disorders [5, 6], and altered BCAA metabolism has been shown to be associated with ASD in an increasing number of studies. However, different studies have shown conflicting and mixed results. For instance, the serum levels of Leu and Val showed a significant decrease in children with ASD compared to neurotypical controls [7, 8], and significant improvements in core symptoms were observed in children with ASD who took BCAA supplements for more than 10 weeks [9]. However, another study showed no significant difference in the levels of BCAAs between children with ASD and healthy controls [10, 11], while yet another showed higher levels of fecal BCAAs in children with ASD compared to controls [12]. In addition, elevated maternal plasma BCAA levels have been associated with increased risk of ASD in children [13]. Similarly, elevated cord plasma BCAA levels have been associated with a greater risk of developing attention deficit hyperactivity disorder in childhood [14]. Therefore, further studies with larger sample sizes are needed to elucidate in detail the relationship between BCAAs and the risk of ASD and the clinical phenotype of ASD.

Dried blood spots (DBSs) are a less invasive method for microvolume blood collection and are an alternative to whole blood samples obtained by venipuncture or arterial sampling. Because they have relatively high stability, require a smaller blood volume, are easy to store and transfer, and reduce the risk of infection, DBSs are already well-established in metabolomic analysis for the high-throughput, reliable, and stable determination of a broad array of analytes and are considered promising for clinical diagnostics and for precision medicine [15].

In our study, we investigated changes in BCAA profiles in DBSs between preschool children with and without ASD in order to provide clues to the etiology of ASD. We selected two cohorts of children with ASD from different hospitals along with typically developing (TD) children from different communities as controls. Moreover, we sought to assess any potential correlation between BCAA profiles and clinical factors within the ASD group.

## Methods

This two-center case-control study protocol was approved by the ethics committee of the Third Affiliated Hospital of Zhengzhou University (Approval #2020-126-01), and informed consent was obtained from all of the primary caregivers.

### Participants

From May 2018 to July 2021, a total of 188 children with ASD from the Center for Child Behavioral Development in two independent tertiary referral hospitals were enrolled consecutively. A total of 144 patients were enrolled from the Third Affiliated Hospital of Zhengzhou University (center 1), and 44 patients were additionally recruited from the Children’s Hospital Affiliated to Zhengzhou University (center 2). The diagnosis of ASD was based on the Diagnostic and Statistical Manual of Mental Disorders, 5th Edition, and the severity of ASD was based on the Childhood Autism Rating Scale (CARS). Children scoring between 30 and 36 were categorized as having mild to moderate ASD, while scores ranging from 37 to 60 indicated severe ASD. The neurodevelopmental levels of children with ASD were evaluated with the Gesell Developmental Schedules (GDS). The GDS includes the five subscales of adaptive behavior, gross motor, fine motor, language, and personal-social. The inclusion criteria were as follows: age between 1 and 6 years; patients having had their anthropometric measurements recorded; and those not having consumed vitamins for more than 3 months prior to the blood sample collection. The exclusion criteria were children having other developmental disorders or psychiatric diseases such as Rett syndrome, cerebral palsy, or chronic seizures; children with hereditary/innate neurological or metabolic diseases; and children with abnormal hepatic or renal function. Over the same time period, 125 TD children from two different communities were recruited as the control group. In addition to meeting the inclusion and exclusion criteria for the ASD group, otherwise healthy children with physical or neurological disorders, systemic medical illnesses, or family histories of any psychiatric disorder were excluded.

All procedures were carried out by trained researchers following international guidelines and regulations. All researchers were doctors, nurses, or researchers with professional medical training. Demographics and anthropometric assessments were carried out by well-trained nurses. The body mass index (BMI) was calculated as kilogram per square meter.

### Preparation of DBS Samples

Venous blood was collected from all of the participants in the morning before breakfast, and a drop of the venous blood was spotted on an absorbent filter paper (Schleicher and Schuell 903 #) to form a DBS. The paper was dried at room temperature and stored at – 20 °C in a polythene bag with a desiccant packet and processed within 2 weeks after sampling.

### Measurement of BCAAs in the DBSs

The DBSs were pretreated using a NeoBase non-derivatized MS/MS kit (PerkinElmer, Wallac Oy, Turku, Finland) and then analyzed using a liquid chromatography-tandem mass spectrometry system (LC-MS/MS) (API3200MD, Applied Biosystems, Foster City, CA, USA) according to a published protocol [16]. Briefly, an area of 3.2 mm diameter was taken from each DBS (equal to 3.2 μL of blood) and placed in a U-96-well microplate, and 100 μL of methanol-containing isotopic internal standards was added to each well. After incubation on a plate shaker for 45 min (45 °C, 700 revolutions/min), 75 μL supernatant was transferred into a fresh V-96-well microplate. To ensure the measurement accuracy, the testing of all samples incorporated blank, low, and high levels of internal quality controls. The levels of Val and Leu were calculated based on the assigned values for the internal standards using ChemoView 2.0.3. Quality control samples were provided by the Centers for Disease Control and Prevention (Atlanta, GA, USA). Total BCAA concentrations were calculated as the sum of Val and Leu.

### Statistical Analyses

Statistical analyses were performed using SPSS version 23 (SPSS Inc., Chicago, IL, USA) and R version 3.6.3 (Foundation for Statistical Computing). Continuous variables were expressed as the mean ± standard deviation (SD) or median values (interquartile range) as assessed by independent group *t*-tests or Mann–Whitney *U*-tests. Categorical variables were expressed as percentages (%) as assessed by chi-squared tests or Fisher’s exact test.

Multivariable regression analysis models were used to assess the correlations between the concentrations of BCAAs in DBSs and the risk of ASD and severity of ASD. Model 1 had no adjustment. Model 2 was adjusted for age, gender, and BMI. Model 3 was adjusted for the same variables as model 2 as well as levels of total BCAAs, Val, and Leu. The trends across the quartiles of BCAAs were tested by treating the quartiles as continuous variables and assigning the midpoint concentrations for each quartile. To further investigate the relationship between BCAA concentrations in DBSs and the risk of ASD and the severity of ASD, restricted cubic spline (RCS) analysis was conducted in Model 3 with the *plotRCS* package in the R software with knots at the 25^th^, 50^th^, and 75^th^ percentiles of the distribution and with the 10^th^ percentile serving as the reference knot.

The three BCAA variables were used to develop the risk prediction model for ASD and were presented as nomograms using the *rms* package in the R software. The performance of the prediction model was evaluated based on its discriminatory ability, calibration ability, and clinical value. The discriminatory ability of BCAAs and the model were evaluated through receiver operating characteristic (ROC) curve analysis and the area under the curve (AUC), and the calibration plot was applied to assess the calibration ability. The model was validated using the bootstrap method with 1000 resamples to quantify any overfitting. A decision curve analysis (DCA) was used to determine the clinical applicability of the nomograms. The R packages *pROC*, *rms*, and *nricens* were used to create all of the graphics.

The Spearman correlation test was used to examine the correlations between BCAA concentrations and the neurodevelopmental level of children with ASD. All statistical tests were two-tailed. Results with *P* < 0.05 and a 95% CI not covering the null value were considered statistically significant.

## Results

### Participant Demographics

A total of 313 participants from two independent centers were enrolled in this study. Among them, 219 children (144 children with ASD and 75 TD children) were enrolled from center 1, and 94 children (44 children with ASD and 50 TD children) were recruited from center 2. The baseline characteristics of the participants are summarized in Table 1. Children with ASD had a younger median age (3.17 years vs. 3.33 years, *P* = 0.021) and higher median BMI values (16.34 vs. 15.51 kg/m^2^, *P* < 0.001) compared to TD children. Of the children with ASD, 54.2% had severe ASD.

**Table 1:** General characteristics of the participants in this study

Variable	ASD (*n* = 188)	Control (*n* = 125)	*P*-value
Age, years	3.17 (2.58, 3.92)	3.33 (3.0, 3.67)	0.021
Male, *n* (100%)	152 (80.9)	105 (84.0)	0.477
BMI, kg/m^2^	16.34 (15.36, 18.00)	15.51 (14.79, 16.44)	< 0.001
Severity of ASD			
Mild to moderate, *n* (100%)	66 (45.8)	-	
Severe, *n* (100%)	78 (54.2)	-	
Total BCAAs (μmol/L)	351.11 (306.92, 402.75)	295.48 (243.33, 355.40)	< 0.001
Val (μmol/L)	183.12 (160.68, 213.63)	161.23 (126.98, 189.79)	< 0.001
Leu (μmol/L)	161.88 (142.98, 196.62)	140.90 (110.40, 175.23)	< 0.001

Data are presented as the number (percentage) for categorical data and as the median (interquartile range) for non-parametrically distributed data. Wilcoxon rank sum tests were used for comparison of the continuous variables according to the data distribution, and chi-square tests were used for the categorical variables. *BCAAs* branched-chain amino acids, *Val* valine, *Leu* leucine/isoleucine, *BMI* body mass index, *ASD* autism spectrum disorder

### Increased Levels of BCAAs Were Seen in the DSBs of Children with ASD

**Fig. 1:**
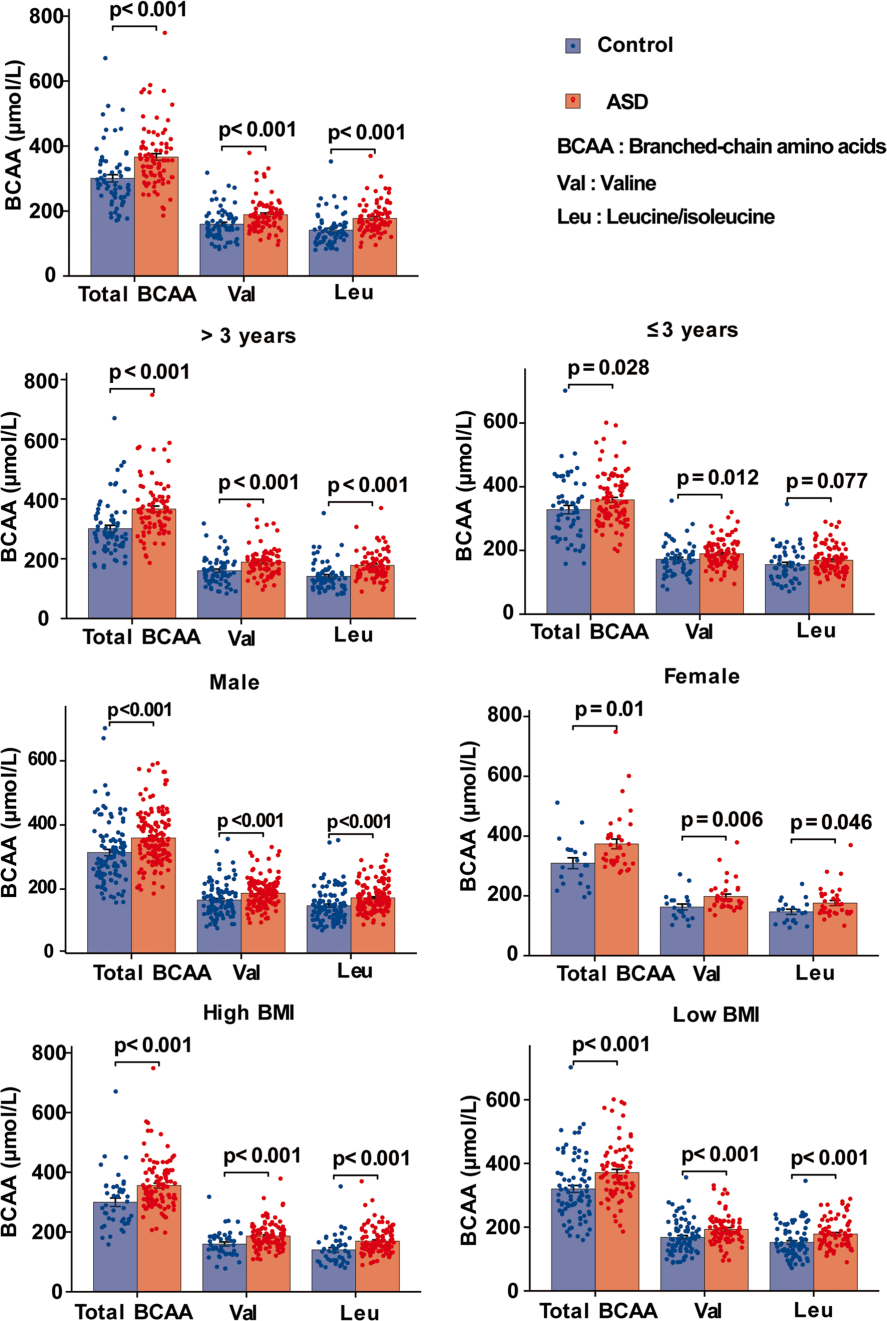
Comparison of BCAA levels between children with ASD and TD controls in different groups stratified by age, gender, and BMI

The levels of total BCAA, Val, and Leu in the children with ASD were higher than those in the control group (*P* < 0.001). Based on the median BMI values, the participants were divided into the low-BMI and high-BMI groups. In the subgroup analyses stratified by gender (male vs. female) and BMI (low BMI vs. high BMI), the children with ASD all exhibited significantly higher BCAAs than TD controls for each covariate (all *P* < 0.05). For the children older than 3 years, the levels of BCAAs in children with ASD were still higher than those in TD children. For the children younger than 3 years old, the levels of total BCAAs and Val were higher in children with ASD compared with TD children, but levels of Leu showed a non-significantly higher tendency in children with ASD (Fig. 1). Thus, clinical factors such as age, gender, and BMI do not impact the increase in BCAA concentrations seen in children with ASD compared with TD controls.

### Associations of BCAAs with the Risk of ASD in Children

We used logistic regression models to analyze the correlation of concentrations of total BCAA, Val, and Leu with ASD in DBSs. After adjusting for gender, age, and BMI, the highest total BCAA levels were associated with an increased risk of ASD (Q4 vs. Q1: adjusted OR = 13.18, 95% CI 5.09–34.10, *P*_trend_ < 0.001). The highest Val levels were associated with an increased risk of ASD (Q4 vs. Q1: adjusted OR = 7.80, 95% CI 3.32–18.34, *P*_trend_ < 0.001), and the highest Leu levels were associated with an increased risk of ASD (Q4 vs. Q1: adjusted OR = 10.85, 95% CI 4.17–28.21, *P*_trend_ < 0.001). Notably, increased levels of total BCAAs (Q4 vs Q1: adjusted OR = 29.84, 95% CI 6.41–183.91, *P*_trend_ < 0.001) remained significantly correlated with ASD even after successively adjusting for Val and Leu levels in model 3. Similarly, increased levels of Val and Leu were still positively associated with the risk of ASD in children in model 3 (Table 2).

**Table 2:** Odds ratios (95% CI) for the risk of ASD associated with quartiles of BCAAs in dried blood spots

Variables	ASD/control	Model 1		Model 2		Model 3	
	*n* (%)	OR (95% CI)	*P*-value	OR (95% CI)	*P*-value	OR (95% CI)	*P*-value
Total BCAA (μmol/L)							
Q1 (< 243.33)	9 (6.3)/19 (25.3)	1.00		1.00		1.0	
Q2 (243.33–295.48)	45 (31.3)/19 (25.3)	3.88 (1.48, 10.16)	< 0.001	3.67 (1.35, 9.96)	0.011	4.60 (1.60, 13.21)	0.005
Q3 (295.48–355.40)	38 (26.4)/19 (25.3)	9.00 (3.57, 22.72)	< 0.001	8.26 (3.16, 21.60)	< 0.001	12.77 (4.10, 39.78)	< 0.001
Q4 (> 355.40)	52 (36.1)/18 (24.0)	12.86 (5.14, 32.14)	< 0.001	13.18 (5.09, 34.10)	< 0.001	29.84 (6.41, 183.91)	< 0.001
*P* trend		< 0.001		< 0.001		< 0.001	
Val (μmol/L)							
Q1 (< 126.98)	14 (9.7)/19 (25.3)	1.00		1.00		1.00	
Q2 (126.98–161.23)	43 (29.9)/19 (25.3)	3.58 (1.52, 8.43)	0.003	3.08 (1.27, 7.49)	0.013	3.31 (1.26, 8.71)	0.015
Q3 (161.23–189.79)	31 (21.5)/19 (25.3)	6.10 (2.65, 14.04)	< 0.001	5.72 (2.41, 13.61)	< 0.001	6.22 (2.06, 18.81)	0.001
Q4 (> 189.79)	56 (38.9)/18 (24.0)	8.0 (3.51, 18.25)	< 0.001	7.80 (3.32, 18.34)	< 0.001	8.95 (1.95, 41.12)	0.005
*P* trend		< 0.001		< 0.001		0.003	
Leu (μmol/L)							
Q1 (< 110.40)	3 (2.1)/19 (25.3)	1.00		1.00		1.00	
Q2 (110.40–140.90)	36 (25.0)/19 (25.3)	4.43 (1.70, 11.51)	0.002	4.38 (1.63, 11.77)	0.003	3.80 (1.32, 10.92)	0.013
Q3 (140.90–175.23)	64 (44.4)/19 (25.3)	10.86 (4.33, 27.26)	< 0.001	10.71 (4.12, 21.80)	< 0.001	8.17 (2.50, 26.77)	0.001
Q4 (> 175.23)	41 (28.5)/18 (24.0)	10.43 (4.15, 26.21)	< 0.001	10.85 (4.17, 28.21)	< 0.001	6.32 (1.16, 34.43)	0.033
*P* trend		< 0.001		< 0.001		0.003	

*P*-values were calculated by logistic regression. Model 1, not adjusted. Model 2, adjusted for sex, age, and BMI. Model 3, adjusted for sex, age, BMI, and levels of total BCAAs, Val, and Leu. *BCAAs* branched-chain amino acids, *Val* valine, *Leu* leucine/isoleucine

### Dose-Response Associations of BCAAs with the Risk of ASD in Children

**Fig. 2:**
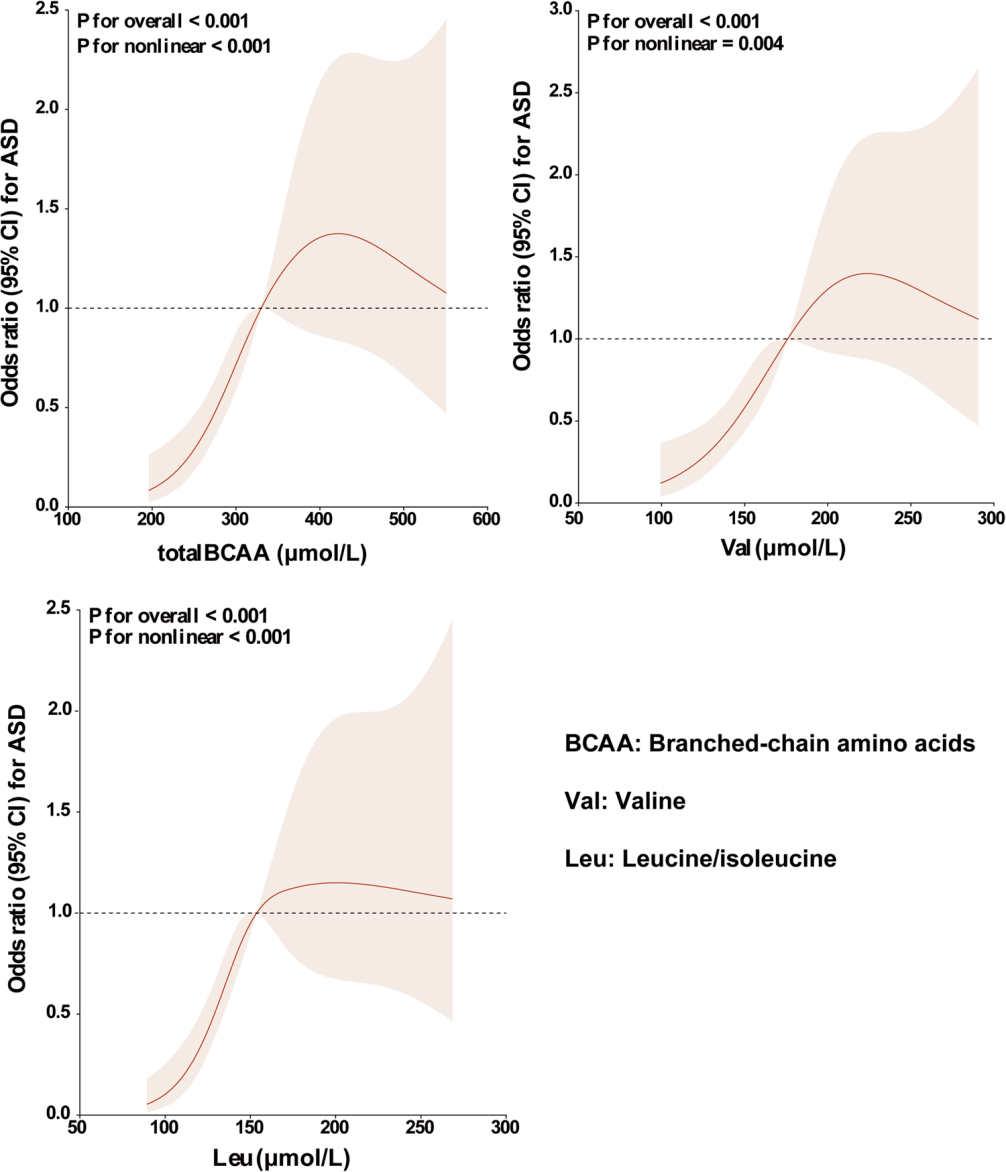
Restricted cubic spline model of the odds ratios of ASD with BCAA levels in DBSs. The red line and red shadow represent adjusted odds ratios and 95% confidence intervals, respectively. The black dotted line represents odds ratios = 1.00. The risk estimates for ASD were adjusted to account for age, gender, BMI, and the levels of total BCAAs, Val, and Leu

The results of RCS regression showed that total BCAA concentrations in DBSs were non-linearly associated with the risk of ASD (*P* for overall < 0.001, *P* for non-linearity < 0.001). Val concentrations in DBSs also showed a non-linear association with the risk of ASD (*P* for overall < 0.001, *P* for non-linearity = 0.004). A similar dose-response relationship was found between Leu concentrations and the risk of ASD (*P* for overall < 0.001, *P* for non-linearity < 0.001) (Fig. 2).

### Construction of the Risk Prediction Model for ASD Using Nomograms

**Fig. 3:**
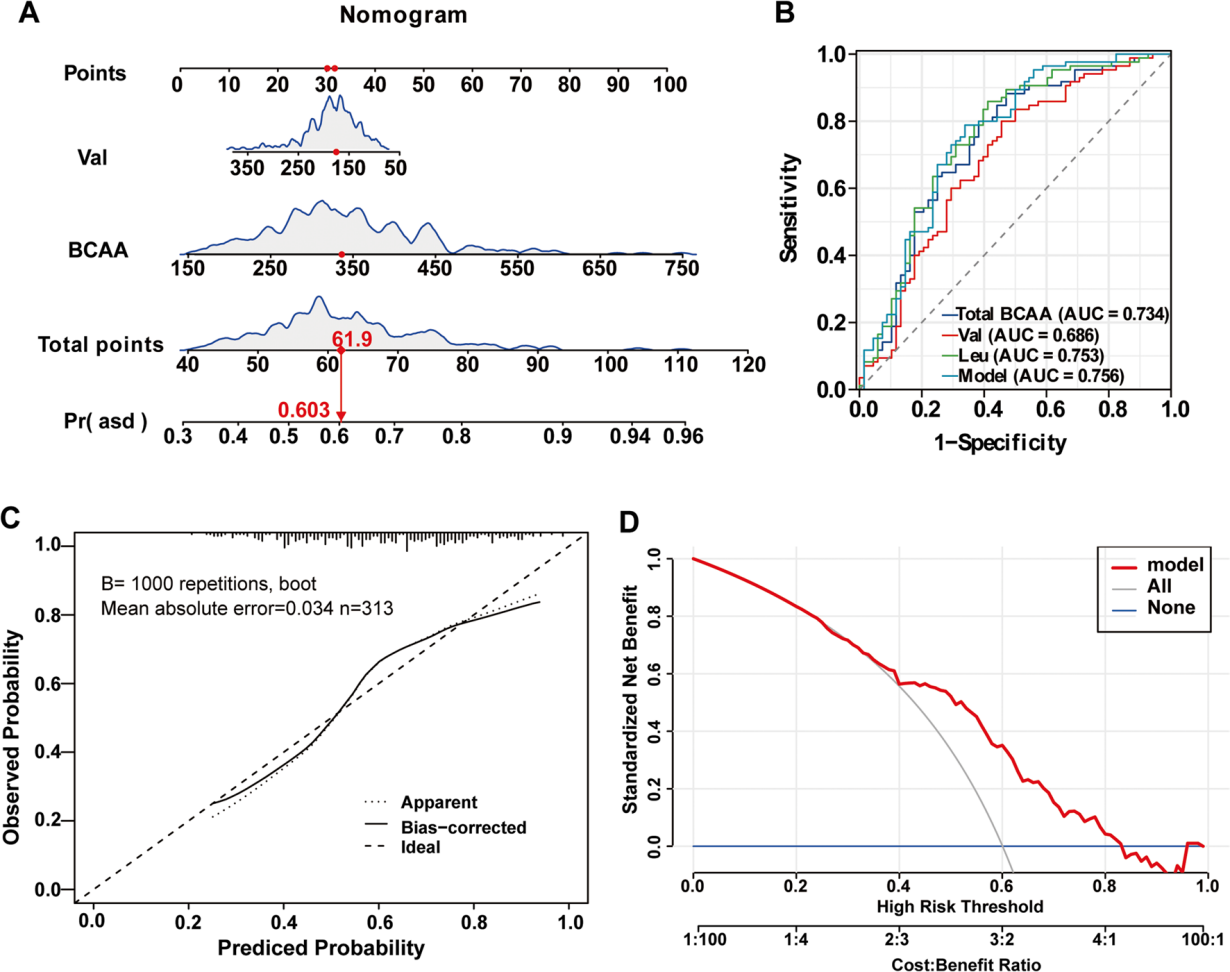
Nomogram for predicting the risk of ASD and model evaluation. **A** A nomogram was constructed based on two independent predictors, and the values of the included factors are marked on the corresponding axis. A vertical line was drawn from the value to the top lines to get the corresponding points. The points identified on the points scale for each variable were summed to obtain the probability of ASD. **B** ROC curves for evaluating the model’s discriminatory ability. **C** Calibration plot of the risk nomogram. The ideal outcome (dashed line), the observed outcome (fine dashed line), and the bias-corrected outcome (solid line) are depicted. **D** DCA for the risk nomogram. The *y*-axis shows the net benefit. The grey solid line represents the assumption that all patients had ASD. The blue solid line represents the assumption that none of the patients had ASD. The red solid line represents the risk nomogram

The three variables of BCAAs (total BCAAs, Val, and Leu) identified by multivariate regression analysis were applied to establish the risk model and presented with a nomogram (Fig. 3A). Total points based on the sum of the points for each predictor in the nomogram were associated with the risk of ASD. Due to the multicollinearity of Leu with total BCAAs and Val, Leu was excluded from the combined model.

The ROC analysis was used to determine the discriminatory ability of the combined nomogram model and single BCAAs. The combined model achieved a higher diagnostic accuracy with an AUC of 0.756 as compared to a single parameter (Val or Leu or total BCAA). The sensitivity and specificity of the nomogram model in recognizing ASD were 78.82% and 66.18%, respectively. As shown in Fig. 3B and Table 3, a total BCAA cut-off level of 283.26 μmol/L was associated with an 88.24% sensitivity and 52.94% specificity (AUC 0.734) by ROC curve analysis, a Val cut-off level of 153.58 μmol/L was associated with an 80% sensitivity and 54.41% specificity (AUC 0.686), and a Leu cut-off level of 138.17 μmol/L was associated with an 85.88% sensitivity and 58.82% specificity (AUC 0.753). Furthermore, the calibration plot (Fig. 3C) of the prediction model showed good consistency between the predicted probability and the actual probability. In the DCA, the area under the model curve, the “grey line,” and the “blue line” represented the clinical effectiveness of the model. The grey solid line represented the assumption that all patients had ASD. The blue solid line represented the assumption that none of all the patients had ASD. The farther the model curve was from the grey and blue lines, the higher the clinical application value of the nomogram. The results of the DCA showed that the net benefits of the nomogram model were significantly higher than the two extreme cases, and the model had a good net benefit for the identification of ASD. The net benefit was greater than 0 when the risk threshold probability was between 0.30 and 0.85 (Fig. 3D).

**Table 3:** Diagnostic accuracy of BCAAs for ASD

	Total BCAAs (μmol/L)	Val (μmol/L)	Leu (μmol/L)	Model
Cut-off value	283.26	153.58	138.17	-
AUC (95% CI)	0.734 (0.651–0.817)	0.686 (0.599–0.773)	0.753 (0.673–0.834)	0.756 (0.676–0.835)
Sensitivity (%)	88.24	80.0	85.88	78.82
Specificity (%)	52.94	54.41	58.82	66.18
PPV (%)	70.09	68.69	72.28	74.44
NPV (%)	78.26	68.52	76.92	71.43
*P*-value	< 0.001	< 0.001	< 0.001	0.001

*BCAAs* branched-chain amino acids, *AUC* area under the curve, *CI* confidence interval, *NPV* negative predictive value, *PPV* positive predictive value, *Val* valine, *Leu* leucine/isoleucine. Model, the combined nomogram model of BCAAs

### The Influence of Age, Gender, and BMI on the Discriminability of BCAAs Between Children with ASD and TD Controls

**Fig. 4:**
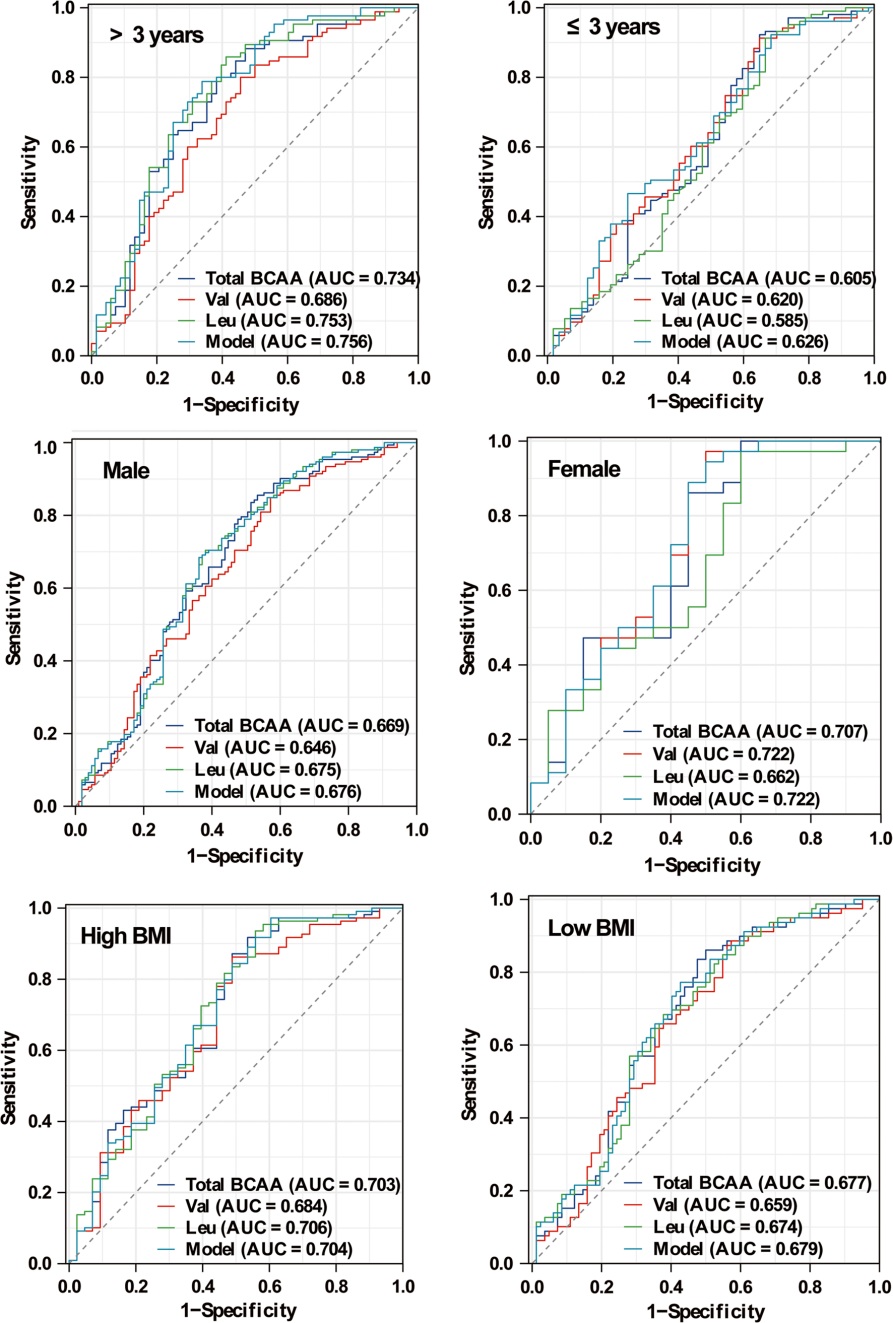
The potential diagnostic value of BCAAs for ASD in the different groups stratified by age, gender, and BMI. Model: the nomogram model constructed with BCAAs

As shown in Fig. 4, the diagnostic ability was analyzed by ROC analyses for the ASD vs. TD groups separately by age, by gender, and by BMI. Compared with boys (AUC 0.676), the discrimination of the model achieved a higher diagnostic accuracy for girls with an AUC of 0.722. Compared with children younger than 3 years old (AUC 0.626), the discrimination of the model achieved a higher diagnostic accuracy for children older than 3 years old with an AUC of 0.756. Compared with children with low BMI (AUC 0.679), the discrimination of the model achieved a higher diagnostic accuracy for children with high values of BMI with an AUC of 0.704. The results showed that the discriminability of the model was much higher for children older than 3 years, suggesting that it could be useful in screening and targeting children older than 3 years who have increased levels of BCAAs and thus are at high risk of ASD.

### The Effect of Clinical Severity on the Levels of BCAAs in Children with ASD

**Fig. 5:**
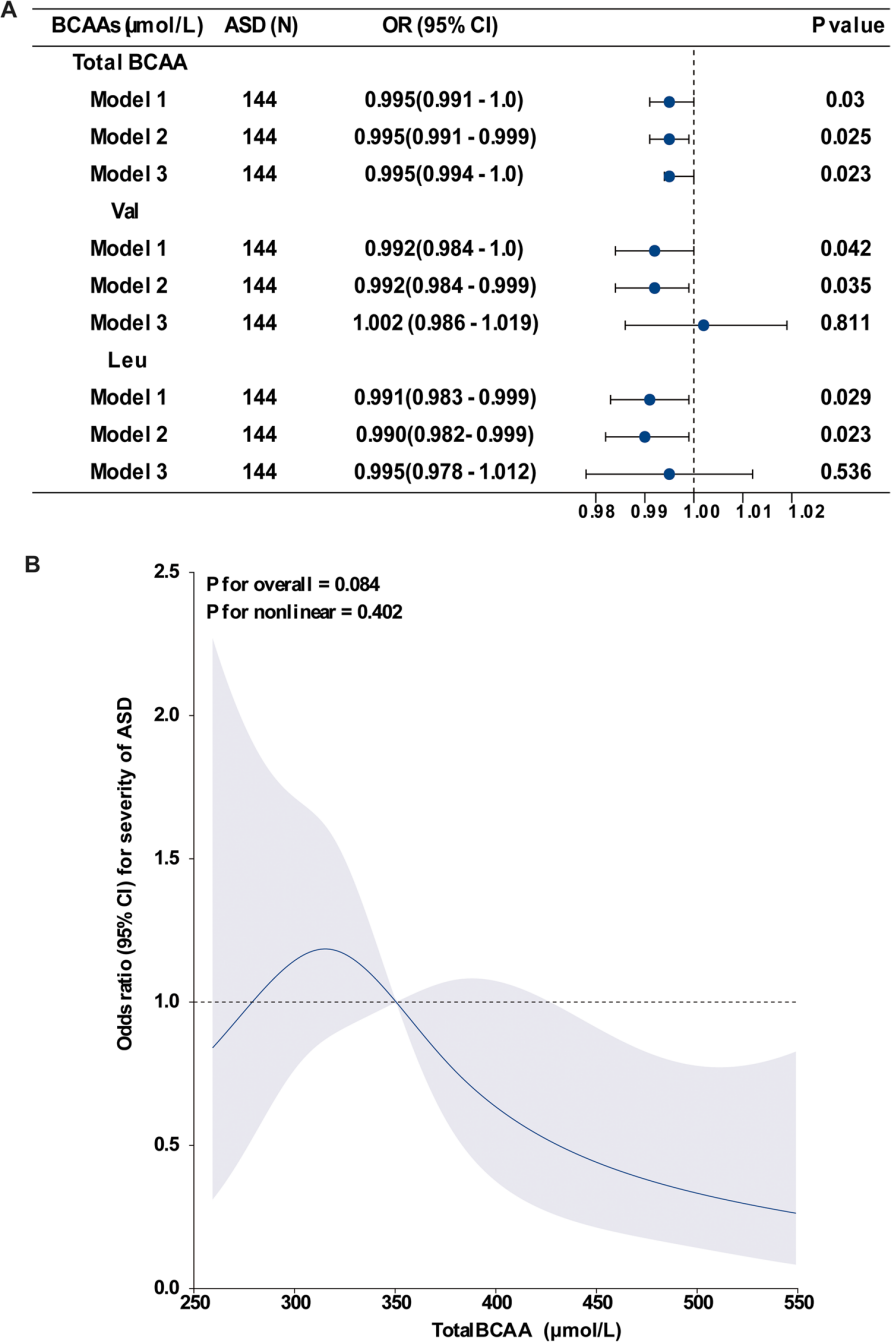
Association of concentrations of BCAAs in DBSs with severity of ASD. **A** Multiple logistic regression analysis of the associations between concentrations of BCAAs and severity of ASD. Model 1, not adjusted. Model 2, adjusted for age, gender, and BMI. Model 3, adjusted for age, gender, BMI, and levels of total BCAAs, Val, and Leu. The blue dots represent the adjusted OR, and the horizontal lines represent the 95% CI. **B** Restricted cubic spline model of the odds ratios of severity of ASD with total BCAA levels in DBSs. The blue line and blue shadow represent adjusted odds ratios and 95% confidence intervals, respectively. The black dotted line represents odds ratios = 1.00

First, we compared the concentrations of BCAAs in DBSs grouped by severity of ASD symptoms in children with ASD. Because no clinical symptoms were collected in children with ASD from center 2, only the baseline demographic and laboratory characteristics of children with ASD in center 1 grouped by the severity of ASD symptoms according to CARS are shown in Table 4. There were 66 children showing mild to moderate ASD symptoms and 78 children showing severe symptoms, and no significant differences were observed for age, gender, or BMI values between the groups of ASD severity. The median values of total BCAA and Val levels in DBSs showed a slight decrease in children with severe symptoms compared with children with mild symptoms (mild to moderate ASD vs. severe ASD: total BCAA 358.74 μmol/L vs. 333.61 μmol/L, *P* = 0.033; Val 195.28 μmol/L vs. 179.09 μmol/L, *P* = 0.039), but these were still higher than those in the group of TD controls, as shown in Table 1, while the median values for Leu levels in DBSs showed no significant decreases between the groups of ASD severity (166.10 μmol/L vs. 152.63 μmol/L, *P* = 0.056). Logistic regression models were used to analyze the relationship between the concentrations of total BCAAs, Val, and Leu in DBSs and the severity of ASD. The results showed that only concentrations of total BCAAs were significantly and negatively correlated with the severity of ASD even after successively adjusting for Val and Leu levels in model 3 (adjusted OR = 0.995, 95% CI 0.994–1.0, *P* = 0.023). The negative relationship between Val and Leu and the severity of ASD disappeared in model 3 (Fig. 5A). Furthermore, RCS regression analysis showed that concentrations of total BCAA in DBSs showed a non-significantly linear association with the severity of ASD (*P* for overall = 0.084, *P* for non-linearity = 0.402) (Fig. 5B).

**Table 4:** Effect of the severity of ASD symptoms on concentrations of BCAAs in children with ASD

	Mild to moderate ASD (*n* = 66)	Severe ASD (*n* = 78)	*P*-value
Age, years	3.13 (2.65, 3.86)	3.00 (2.42, 4.27)	0.729
Male, *n* (100%)	116 (80.6)	63 (84.0)	0.944
BMI, kg/m^2^	16.50 (15.67, 18.51)	16.42 (15.23, 17.56)	0.292
Total BCAAs (μmol/L)	358.74 (316.0, 432.34)	333.61 (301.37, 391.03)	0.033
Val (μmol/L)	195.28 (169.18, 284.53)	179.09 (160.0, 210.01)	0.039
Leu (μmol/L)	166.10 (145.94, 204.99)	152.63 (141.54, 176.92)	0.056

Data are presented as the number (percentage) for categorical data and as the median (interquartile range) for non-parametrically distributed data. Wilcoxon rank sum tests were used for comparison of the continuous variables according to the data distribution, and chi-square tests were used for the categorical variables. *BCAAs* branched-chain amino acids, *Val* valine, *Leu* leucine/isoleucine, *BMI* body mass index, *ASD* autism spectrum disorder

Second, the correlation analyses between concentrations of BCAAs and neurodevelopmental developmental quotient (DQ) scores were examined, and there was no obvious correlation between BCAAs with the GDS DQs of adaptive behaviors, gross motor, fine motor, language, or personal-social (all *P* > 0.05).

## Discussion

Over the past decade, there have been a few studies showing a link between BCAAs and ASD in children, but the conclusions are inconsistent due to the limited sample sizes of these studies. Although some of the studies reported a reduction of BCAA levels in children with ASD, further research is warranted. Our study revealed that concentrations of BCAAs were higher in children with ASD compared with TD children. The concentrations of BCAAs were positively associated with the risk of ASD, and clinical profiles did not affect the association between BCAAs and the risk of ASD. Meanwhile, total BCAAs, Val, and Leu concentrations in DBSs were non-linearly associated with risk of ASD, which partly explained the inconsistency of our findings. The developed nomogram model could be used as a diagnostic biomarker for ASD, especially for children older than 3 years. Moreover, total BCAA concentrations of total BCAAs were slightly negatively associated with the severity of ASD.

While most current research on the relationship between BCAAs and ASD has shown reduced BCAA levels in cases versus controls, prior animal studies in rats have shown that BCAA administration promotes neurological dysfunction such as epilepsies [17], maple syrup urine disease (MSUD), and neurobehavioral impairment [18]. BCAAs are minimally metabolized by the liver, and blood BCAAs can readily cross the blood–brain barrier; thus, oral ingestion of BCAAs can lead to a transient increase in BCAA levels in the brain. Based on these prior findings, we hypothesized that increased BCAAs in the brain are associated with an increased risk of ASD, which was confirmed by the results of our study. The inconsistency of the findings on the relationship between BCAAs and risk of ASD may be due to the heterogeneity of the study population or to the non-linear relationship between BCAA concentrations and the risk of ASD. Therefore, we used RCS regression to analyze the dose-response relationship between BCAA concentration and risk of ASD, and our results showed that BCAA concentrations were all non-linearly associated with the risk of ASD.

While the exact mechanisms underlying the role of BCAAs in children with ASD remain to be investigated, several possibilities have been raised in the literature. One of these possibilities is that elevated BCAA concentrations might affect protein synthesis, energy production, and neurotransmitter balance. The accumulation of BCAAs, especially Leu, has been shown to cause an imbalance in other essential amino acids and to cause neurotransmitter depletion [19], including glutamate. Such accumulation has also been shown to reduce dopamine and serotonin tone, which tend to be lower in ASD patients, and intranasal treatment with dopamine or serotonin can effectively rectify ASD-like behavioral phenotypes in rats [20–22]. Increased BCAA catabolism indicates dysfunction of mitochondrial beta oxidation, which can overload mitochondria and lead to defective energy metabolism [23]. Beta oxidation in mitochondria is a precursor to mitochondrial dysfunction, which is more prevalent in individuals with ASD and is thought to be linked to the etiology of ASD [24].

Another possible mechanism by which BCAAs are involved in the pathogenesis of autism is that excess levels of BCAAs can lead to neurological damage. On the one hand, toxic accumulation of BCAAs in the brain induces the overproduction of reactive oxygen species and increases autophagy and apoptosis in glial cells and neurons, which might be potential pathogenic mechanisms in patients with MSUD [25–27]. On the other, high levels of BCAAs result in an increase in brain-derived neurotrophic factor, impaired spatial memory, and cognitive impairment [18]. In addition, circulating plasma BCAA exposure was found to be positively associated with ischemic stroke, and the pathophysiology was argued to be that BCAAs (Leu and Val) aggravated glia-induced neuroinflammatory responses in the brain [28]. Altered BCAA metabolism has also been linked to other neurological and mental health disorders. For example, elevated blood levels of BCAAs have been reported in patients with Alzheimer’s disease [29], and long-term chronic oral BCAAs are associated with neuron loss and seizures [30]. Supplementation with BCAAs in mice resulted in anxiety-like behavior, which is a common characteristic in several neurocognitive disorders [31]. Treatment for major depressive disorder was shown to be more effective in individuals with lower BCAA concentrations, thus allowing for the prediction of response to treatment using metabolic profile analysis [27]. Taken together, these studies suggest that higher BCAA levels may be associated with a variety of neurodevelopmental disorders, and thus, it is plausible to conclude that BCAAs can contribute to ASD. Because BCAAs can be supplemented from the diet, our findings suggest that there may be a subset of ASD cases that are treatable by simple dietary interventions.

Our findings from this study indicate that increased BCAAs in DBSs are positively associated with ASD and may serve as a stable biomarker for distinguishing children with ASD from TD subjects. Due to the non-invasive and easy-to-use advantages of DBSs, it is reasonable to promote such an approach in clinical practice. The finding that concentrations of BCAAs were higher in children with ASD was robust after adjustment for relevant covariates, and the differences in BCAA levels between children with ASD and TD controls were stable and were not changed by clinical characteristics such as age, gender, or BMI. In addition, we observed an age difference in the diagnostic ability of BCAAs for discriminating children with ASD from TD controls, and the combined nomogram model yielded good predictive values for children older than 3 years. None of the aforementioned studies performed an age-specific diagnostic analysis, so further studies are necessary to confirm this preliminary finding of the predictive ability of BCAAs. Thus, the combined model of BCAAs might be used to identify a biologically homogeneous subgroup of ASD patients, predict the response to treatments and adverse reactions to medications, and assist in the development of novel drugs that target specific core symptoms of ASD.

Within the small-scale population of children with ASD, we observed a slightly negative and linear correlation between concentrations of total BCAAs and the severity of ASD; that is, concentrations of total BCAAs were slightly lower in cases of severe ASD than mild to moderate ASD. On the one hand, the difference in concentrations of BCAAs grouped by severity of ASD could possibly be influenced by dietary and lifestyle factors [32]. On the other, despite the reduction in BCAA concentrations in cases of severe ASD, they still had higher concentrations of BCAAs than TD controls. Consistent with the findings of the abovementioned dose-response analysis of total BCAAs and the risk of ASD, above a certain concentration, total BCAAs showed a negative association with the risk of ASD and severity of ASD. As far as we know, there are only a few clinical studies on levels of BCAAs and the clinical severity of ASD, and further research is needed. In addition, our results did not find any relationship between the concentrations of BCAAs and neurodevelopmental scales scores in children with ASD, and thus, further clinical studies are also needed to investigate the effect of concentrations of BCAAs on neurodevelopment in children with ASD.

This study is not without limitations. First, we did not record the diet in children with ASD and therefore cannot state whether the increases in BCAAs were diet-related. Because dietary factors were not excluded from our study, our findings should be regarded as hypothesis-generating and warrant further investigation and confirmation. Second, despite the strong association of BCAAs with the gut microbiome [33], further subgroup analyses could not be performed because our data lacked detailed information on gastrointestinal comorbidities. This is an important issue that will need to be resolved in future studies. Furthermore, future research endeavors will be essential to complete and analyze the association between BCAAs and ASD along with other comorbidities like epilepsy and sleep disorders. Third, current assays are unable to distinguish leucine from isoleucine, so our study could not clarify the relationship between the two amino acids and the risk of ASD. In addition to human studies, animal studies can help to resolve the pathogenic role of excess BCAAs in ASD and the mechanisms behind this.

## Conclusions

In this two-center case control study, we found that concentrations of BCAAs in the DBSs were associated with increased risk of ASD, and the association was not affected by gender, age, or BMI. Moreover, BCAAs were non-linearly associated with the risk of ASD. The combined model of BCAAs had the ability to diagnose children at high risk of ASD, potentially allowing for early prediction and prevention. Further studies on the effects of co-morbidities such as gastrointestinal disorders, diet, and other factors on BCAA concentrations might more clearly elucidate the relation between BCAAs and the risk of ASD.

## Data Availability

Datasets generated during and/or analyzed during the current study will be made available from the corresponding author on request.
